# Transcription Factor Binding Site Redundancy in Embryonic Enhancers of the *Drosophila* Bithorax Complex

**DOI:** 10.1534/g3.111.001404

**Published:** 2011-12-01

**Authors:** Robert A. Drewell

**Affiliations:** Biology Department, Harvey Mudd College, Claremont, California 91711

**Keywords:** *Drosophila*, bithorax complex, *cis*-regulation, enhancer, transcription factor, DNA binding site

## Abstract

The molecular control of gene expression in development is mediated through the activity of embryonic enhancer *cis*-regulatory modules. This activity is determined by the combination of repressor and activator transcription factors that bind at specific DNA sequences in the enhancer. A proposed mechanism to ensure a high fidelity of transcriptional output is functional redundancy between closely spaced binding sites within an enhancer. Here I show that at the bithorax complex in *Drosophila* there is selective redundancy for both repressor and activator factor binding sites *in vivo*. The absence of compensatory binding sites is responsible for two rare gain-of-function mutations in the complex.

Enhancer *cis*-regulatory modules are regions of nonprotein coding genomic DNA that bind protein transcription factors (TFs) to direct expression of target genes (Arnone and Davidson 1997; Borok *et al.* 2010). Transcriptional regulation by enhancers is fundamental to embryonic development and evolutionary diversity in metazoans (Levine and Tjian 2003; Wittkopp 2010; Wray 2007). The *Drosophila melanogaster* bithorax complex (BX-C) provides a tractable model system in which to dissect the functional activities of embryonic enhancers. The BX-C is a 330-kb genomic region (Martin *et al.* 1995) that contains just three homeotic genes (Lewis 1978). Expression of these genes is controlled by numerous enhancers arranged in the *infraabdominal* (*iab*) intergenic regions (Celniker *et al.* 1990), which regulate the spatial and temporal expression of the homeotic genes along the anteroposterior axis of the developing embryo (for detailed reviews, see Akbari *et al.* 2006; and Maeda and Karch 2006). The activity of the BX-C embryonic enhancers is controlled by TFs expressed at the earliest stages of development (Busturia and Bienz 1993; Ho *et al.* 2009; Zhou *et al.* 1999). The TFs form input signals by recognizing and binding in a sequence-specific manner in the enhancer DNA at transcription factor binding sites (TFBS). Once bound, TFs mediate the transcriptional output of the enhancer in a number of ways, including interactions with the basal transcriptional machinery at the promoter of the target gene which helps recruit RNA polymerase II (activators) (Kadonaga 2004) or by preventing the binding of additional protein factors at closely located binding sites (short-range repressors) (Small *et al.* 1991). Activators can act over very large genomic distances (>50 kb) (Ho *et al.* 2011), whereas short-range repression appears to be limited to distances of approximately 100 bp and certainly less than 400 bp (Kulkarni and Arnosti 2005). For example, the IAB5 enhancer in the BX-C is activated by the pair-rule TF FUSHI-TARAZU (FTZ) but is repressed by the gap TFs KRUPPEL (KR) and HUNCHBACK (HB) (Figure 1A) (Busturia and Bienz 1993; Ho *et al.* 2009; Starr *et al.* 2011).

**Figure 1:**
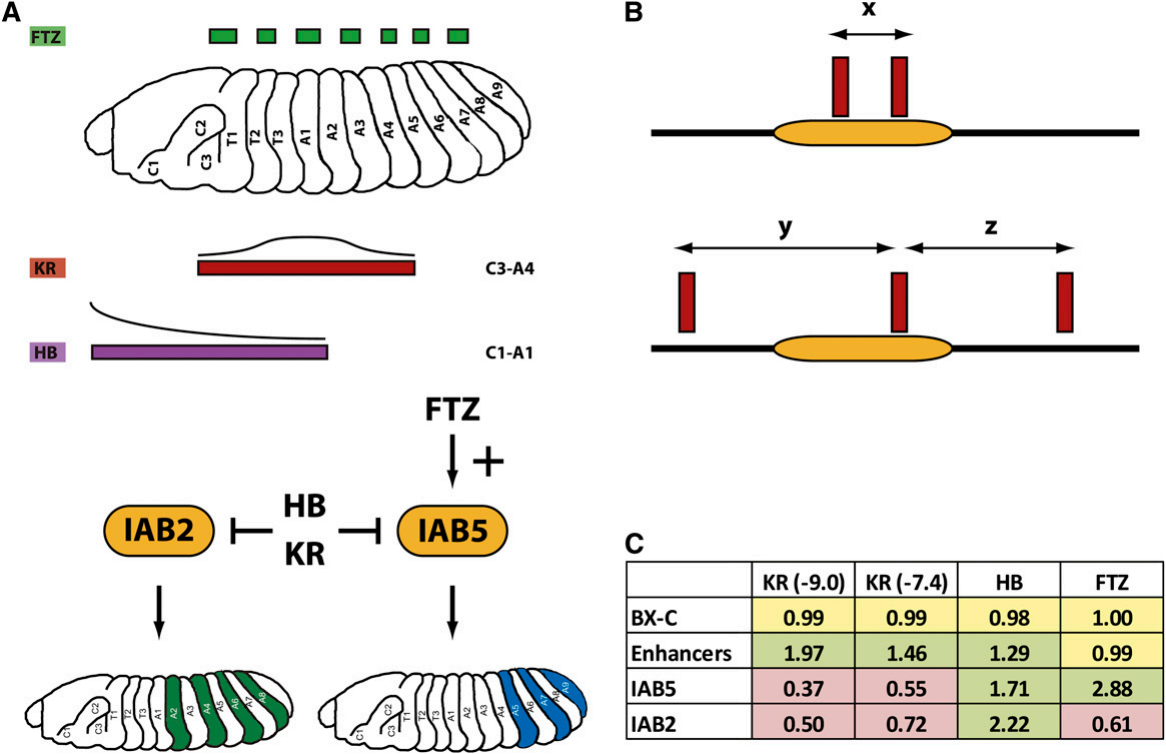
(A) The regulatory output of the IAB5 and IAB2 enhancers is determined by specific TF inputs. The pair-rule TF FUSHI-TARAZU (FTZ) acts as an activator of IAB5 in alternating body segments of the embryo, whereas KRUPPEL (KR) and HUNCHBACK (HB) act as repressors at the BX-C enhancers in broad regions of the embryo. The activator for IAB2 is currently unknown. (B) Model of TFBS redundancy at an enhancer (orange rectangle). In the upper panel, the distance between two neighboring binding sites (x) is close enough so that the loss of one site can be functionally compensated for by the adjacent site. In the lower panel, the distances to the neighboring sites (y and z) are too great to allow functional redundancy. (C) The calculated ratio of TFBS spacing for the entire BX-C (excluding all enhancers); the IAB8, IAB7, and IAB6 enhancers grouped together (Enhancers); IAB5 and IAB2 for KR (at high stringency [ln(p) < −9.0] and low stringency [ln(p) < −7.4]), HB, and FTZ are shown. A value >1 indicates that binding sites are closer together, and a value <1 indicates that sites are more distantly spaced relative to the expected spacing (= size of the entire BX-C/total number of binding sites).

Because gene expression must be tightly regulated to allow normal embryonic development, then it follows that the recruitment of specific TFs to enhancers should be very robust. A critical molecular mechanism to ensure this robustness is selection for clusters of functional binding sites (Berman *et al.* 2002; Berman *et al.* 2004), as evidenced by the evolutionary conservation of TFBSs at enhancers in the BX-C (Ho *et al.* 2009; Starr *et al.* 2011) and at the *even-skipped* gene (Crocker and Erives 2008; Hare *et al.* 2008b; Ludwig *et al.* 1998) in divergent insect species. An additional potential mechanism yet to be fully explored is the extent to which clustering is responsible for functional redundancy between binding sites (Figure 1B). The key question is whether redundancy of TFBSs is a common theme in embryonic enhancers. One way to answer this question is to investigate examples of sequence mutations that result in disruption of a TFBS by examining the functional consequences for enhancer activity. However, during 30 years of intensive molecular analysis of the 330 kb of the BX-C, only two such mutations have ever been identified; the *Superabdominal* (*Sab*) mutation in the IAB5 enhancer (Celniker *et al.* 1990) and the *Hyperabdominal* (*Hab*) mutation in the IAB2 enhancer (Lewis 1978). In both cases the loss of a KR short-range repressor binding site permits the enhancer to respond to an input signal from an activator in ectopic embryonic segments (Ho *et al.* 2009; Shimell *et al.* 1994). Why are there so few mutations in the BX-C that disrupt enhancer function? The discovery of only two gain-of-function point mutations in the entire complex suggests that there may be extensive functional redundancy between repressor binding sites at the enhancers. The aim of this study is to investigate the extent of TFBS clustering in the BX-C and address the implications for binding site redundancy and enhancer function.

## Material and Methods

### Genomic sequences

**Figure 2:**
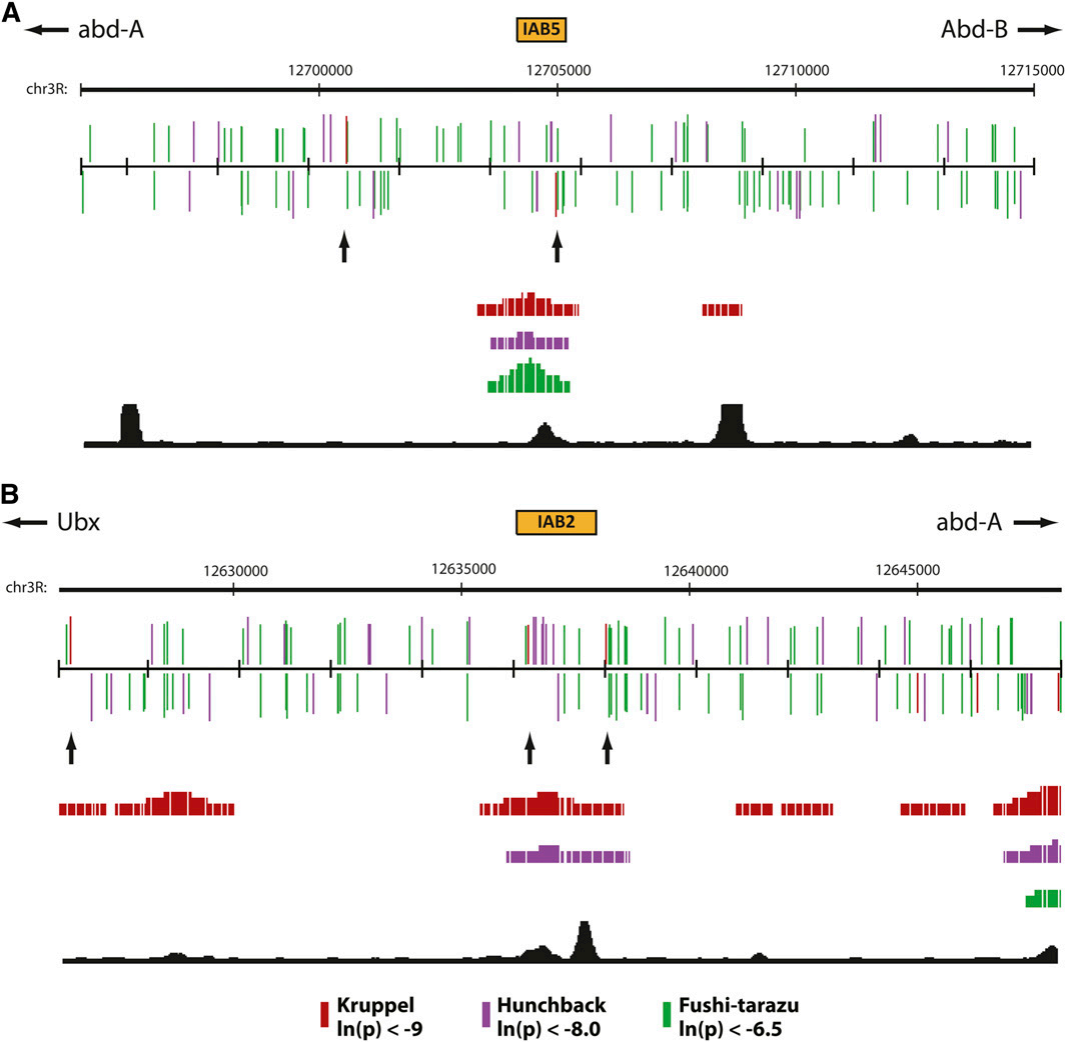
(A) IAB5 and (B) IAB2 enhancers (orange boxes) and surrounding 20-kb genomic regions are shown as a custom track in the UCSC Genome Browser. PATSER was used to predict the spatial distribution of binding sites on the forward (top) and reverse (bottom) DNA strands for KRUPPEL (KR, red), HUNCHBACK (HB, purple), and FUSHI-TARAZU (FTZ, green). Rectangle height is proportional to the score strength of each predicted TF binding site. KR binding sites in the enhancer and neighboring sites are indicated with arrows. The Berkeley Drosophila Transcription Network Project ChIP/chip track (Macarthur *et al.* 2009) shows the location of verified *in vivo* binding sites for KR (red), HB (purple), and FTZ (green). The BNTNP chromatin accessibility track (black) identifies DNase I sensitive sites.

Genomic regions from the *Drosophila melanogaster* BX-C from the annotated U31961 sequence were identified in the Berkeley Drosophila Genome Project *D. melanogaster* genome (annotated April 2006 release) on the University of California Santa Cruz (UCSC) Genome Browser (http://www.genome.ucsc.edu) (Kent *et al.* 2002) and shown as “Chr3R” in Figure 2.

### Computational analysis of TFBS

Sequence from the *D. melanogaster* BX-C was analyzed by use of the UCSC Genome Browser as previously described (Ho *et al.* 2009; Starr *et al.* 2011). PATSER (http://rsat.ulb.ac.be/rsat/patser_form.cgi) (Hertz and Stormo 1999; Thomas-Chollier *et al.* 2008) and previously assembled Position Weight Matrices for the three TFs; KRUPPEL (KR), HUNCHBACK (HB), and FUSHI-TARAZU (FTZ) (Ho *et al.* 2009; Starr *et al.* 2011) were used to search for binding sites. ln(p-value) cutoff values for predicted sites were selected according to the values of confirmed functional binding sites as described in previous studies (Hare *et al.* 2008a; Ho *et al.* 2009; Starr *et al.* 2011).

### *In vivo* TF binding

TF binding and DNase I accessibility data were mapped on the BX-C sequence in the UCSC Genome Browser (Kent *et al.* 2002). The Berkeley Drosophila Transcription Network Project ChIP/chip track (Macarthur *et al.* 2009) was used to identify the location of verified binding sites for the KR, HB, and FTZ TFs in stage 4-5 embryos (1% false discovery rate). The BNTNP chromatin accessibility track was used to identify DNase I sensitive sites in stage 5 embryos. The ORegAnno track (Griffith *et al.* 2008) was used to identify the genomic location of the IAB5 and IAB2 enhancers.

## Results and Discussion

If clustering is an important functional feature for TFBS redundancy in embryonic enhancers of the BX-C, then one prediction is that there should be a greater likelihood of finding two binding sites for a particular TF in close proximity to each other in a defined enhancer when compared with the complex as a whole. To address this hypothesis, I analyzed the distribution of KR binding sites across the entire BX-C (supporting information, Table S1). Intriguingly, at high stringency (ln(p) < −9.0) KR binding sites are enriched in the BX-C when compared with randomized sequence generated from the entire BX-C (Table S1 and Table S2, χ^2^ distribution test, *P* = 1.52^−7^). In addition KR sites are highly enriched in the characterized IAB8, IAB7, and IAB6 enhancers compared with the whole of the BX-C (Figure 1C, χ^2^ distribution test, *P* = 4.06^−158^) with an average space between sites of 1499.1 and 2978.5 bp, respectively. Surprisingly, IAB5 and IAB2 are significantly depleted in KR binding sites (Figure 1C), with each enhancer containing only one site corresponding to the functional sites identified in the *Sab* and *Hab* mutations (Ho *et al.* 2009; Shimell *et al.* 1994). In the case of IAB5, the average distance to the neighboring KR sites is 8028 bp, whereas for IAB2 it is 5857.5 bp. For IAB5 and IAB2 no single adjacent KR site is closer than 1702 bp, well beyond the proposed range of action for a short-range repressor (Kulkarni and Arnosti 2005; Li and Arnosti 2011), indicating that in both cases there is no functionally redundant KR site available to compensate for loss of binding at the *Sab* and *Hab* sites (Figure 2). Even when a less-stringent threshold value (ln(p) < −7.4) is used that identifies four times as many putative KR binding sites in the BX-C, the IAB5 and IAB2 enhancers are depleted in sites (Table S3 and Figure 1C). In contrast, the relative abundance of KR sites at the other embryonic enhancers from the BX-C may provide an explanation for the fact that no gain-of-function mutations have ever been characterized in the complex outside of IAB5 and IAB2.

To investigate whether the enrichment of repressor TFBSs in embryonic enhancers extends beyond KR I also examined the spacing of HB binding sites across the BX-C (Table S4). In agreement with the finding for KR, HB sites are found in close proximity in the embryonic enhancers of the complex (Figure 1C, χ^2^ distribution test, *P* = 2.28^−9^). In the case of HB, the IAB5 (*P* = 6.12^−28^) and IAB2 (*P* = 9.59^−48^) enhancers are also highly enriched in binding sites compared with the BX-C as a whole (Figure 1C and 2). This discovery correlates with the absence of any gain-of-function mutations resulting from the loss of HB binding sites in the BX-C and suggests that extensive functional redundancy between the multiple HB sites in each enhancer may exist. Such clustering also appears to extend to FTZ TFBSs in the BX-C (Table S5), because sites for this factor are significantly enriched in the IAB5 enhancer (Figure 1C, χ^2^ distribution test, *P* = 9.02^−26^), for which FTZ is the known activator (Busturia and Bienz 1993), but depleted in IAB2 (χ^2^ distribution test, *P* = 1.01^−25^; Figure 1C), which does not recruit FTZ (Figure 2).

Taken together, the data indicate that extensive functional redundancy exists through clustering for TFBSs in the embryonic enhancers of the BX-C. This is reflected in the close spacing of repressor (KR and HB) and activator (FTZ) binding sites in enhancers that are known to recruit these factors *in vivo* (Figure 2) and offers insight into why so few gain-of-function mutations have ever been discovered in the complex. Furthermore, in the two examples where a point mutation in a TFBS from the BX-C does appear to prevent the functional recruitment of the KR repressor (*Sab* and *Hab*) there is a significant depletion of binding sites in the genomic neighborhood that could potentially compensate for loss of KR binding. It will be of critical interest in future studies to investigate whether similar architectural arrangements of TFBSs exist in other model systems.

## Supplementary Material

Supporting Information
