# Supplementary material for: Transcription Factor Binding Site Redundancy in Embryonic Enhancers of the Drosophila Bithorax Complex
Source: G3 (Bethesda). 2011 Dec 1;1(7):603–6. doi: 10.1534/g3.111.001404 (PMC3276168; doi:10.1534/g3.111.001404)
Supplement: Supporting Information [file supp_1_7_603__index.html]

Supporting Information 

# Transcription Factor Binding Site Redundancy in Embryonic Enhancers of the *Drosophila* Bithorax Complex

## Supporting Information for Drewell, 2011

**Files in this Data Supplement:**

- Table S1 - The position and score of predicted transcription factor binding sites for KRUPPEL at high stringency (ln(p)<-9.0) in the BX-C

  The distance to neighboring binding sites (Dis R/L and Avg dis) is also shown. Sites mapping within the identified IAB8, IAB7 and IAB6 (light green) and IAB5 or IAB2 (light blue) embryonic enhancers are annotated (Anno). The data from the these tables is summarized in Figure 1c.

  (.xls, 60 KB)
- Table S2 - KRUPPEL at high stringency (ln(p)<-9.0) in randomized sequence from the BX-C (.xls, 44 KB)
- Table S3 - KRUPPEL at low stringency (ln(p)<-7.4) in the BX-C (.xls, 140 KB)
- Table S4 - HUNCHBACK in the BX-C (.xls, 164 KB)
- Table S5 - FTZ in the BX-C (.xls, 404 KB)
